# Loot

**Published:** 1870-05

**Authors:** 


### Loot.

### On the Acid Dyspepsia of Infants.

By EUSTACE SMITH, M. D., Physician Extraordinary to His Majesty the King of the Belgians, Physician to the Northwest London Free Dispensary for Sick Children, Etc.

Acid dyspepsia is one of the commonest digestive derangements met with in young children, and few infants can be said to escape it altogether. A trifling complaint, and readily recovered from when attended to early and judiciously treated, if neglected it becomes a most serious and obstinate disorder, which may resist all treatment, and may lead to the most extreme emaciation, or even to death itself.

The food taken seems shortly after being swallowed to undergo an acid fermentation ; sour gases are evolved, great discomfort is produced, and nutrition is seriously interfered with. The derangement is usually caused by over-feeding with farinaceous foods. It is too commonly the case that these foods are given in enormous quantities — in quantities greater than any infant with ordinary digestive power can by any possibility assimilate. The reason ot this reckless feeding is, partly, the mistaken notion which so universally prevails of the digestibility of these foods; partly, the eagerness with which the child himself will swallow large masses of sop; for the griping and flatulence occasioned by the presence of large masses of starchy matters in the alimentary canal will — if not too severe — excite a fictitious hunger which is not easily appeased. An infant of three or four months old, in whom the secretion of saliva is but lately established, or an infant of yet earlier age, who has no saliva at all, is often fed with a large table-spoonfull of corn flour or other farinaceous powder, boiled with milk or with water, four, five, or even more times in the day. The food lies undigested in the bowels, ferments, and a state of acid indigestion is set up, which does not cease with the removal by vomiting and purging of the cause which has produced it. Even a return to a simpler diet is often insufficient by itself to put an end to the derangement; plain milk and water is vomited sour and curdled, and everything taken into the stomach seems to undergo the same acid change.

As this derangement is so easily excited by improper feeding, even in healthy infants, children whose strength has been already reduced by disease, and whose digestive power is therefore lowered in proportion to the weakness of the whole system, are still more likely to be affected by the same cause. On this account acid dyspepsia is a not unfrequent sequel of acute disease in infants, and may, after apparent convalescence from the primary disorder, lead to death, by the interference with nutrition and by the exhaustion which it so often produces. The diarrhœa, which is a not uncommon sequence of some of the acute specific diseases, as scarlatina and measles, is often primarily excited by this derangement, and is too frequently a cause of death. Severe operations upon the child, such as that for stone in the bladder, may also be followed by the same complication, for anything which lowers the easily depressed general strength reduces also the digestive power, and predisposes to this complaint.

Children brought up by hand are especially liable to this acid dyspepsia, for even when fed upon a suitable diet, carelessness in the administration of the food selected, so that the stomach is overloaded by too frequent or too copious meals, or neglect of the necessary cleanliness, so that they are allowed to take milk which by being put into a sour bottle has already begun to change, will excite this indigestion. Amongst the poor of London, it is not uncommon to find a child brought for medical advice sucking at a feeding bottle, of which the intensely sour smell at once discloses the cause and suggests means for the relief of the complaint under which he is laboring.

The earliest symptoms of this derangement are due to the uneasiness produced by flatulent distension and griping pains. The infant is restless and fretful, whining and crying, and refusing to be pacified. Large quantities of gas are evacuated, both by the mouth and the rectum, affording at first some relief, and the child becomes quieter until a re-accumulation takes place. At night the griping is exceedingly distressing, and his sleeplessness at this time by the discomfort it occasions to his attendants, is usually the symptom which assumes the greatest prominence in the mind of the mother, and is the chief reason for applying for advice. The infant, after lying for a time in uneasy sleep, starting, twitching, moaning, frowning, and drawing up the corners of his mouth, suddenly wakes up with a loud cry, and is seized with a fit of violent screaming which resists all efforts to calm him. He throws himself from side to side, jerks about his lower limbs, or suddenly straightening them out in a line with his body, becomes for a few moments rigid as if turned into stone. These attacks of colic are sometimes so severe as to cause great alarm; the child falling into a state of collapse, or being thrown into convulsions, which may be repeated again and again. The ravenous appetite noticed in children suffering from flatulence has already been referred to. This symptom usually disappears as the derangement becomes more marked. Vomiting comes on after a time, the appetite then fails, and the child is thirsty and feverish. Vomiting is at first excited by taking food, but may afterwards occur when no food has been lately taken, and in bad cases may be caused by a sudden movement, or even by a touch, as in wiping the mouth. The vomited matters consist at first of food and curdled milk, afterwards of clear fluid like water; the smell is usually intensely sour. The bowels at first are confined, but after a time diarrhœa comes on, the motions being either pale, frothy, and sour-smelling, or watery and fetid. There may be straining during the passage of a stool, in which case the motions may contain streaks of blood. An eruption of red strophulus, covering the body and arms of the child, is a not uncommon symptom; it may be mixed with urticaria.

An infant suffering from this derangement soon becomes pale and thin. His face assumes a constant expression of fretfulness, which is increased by the furrow which appears, passing on each side from the nose, to encircle the corner of the mouth. The lower eyelid and upper lip are disposed to be livid; the lips twitch, and the corners of the mouth are frequently drawn up, giving a peculiarly plaintive and helpless expression to the face. The fontanelle is depressed more or less deeply, according to the degree to which the strength is reduced. The eyes sometimes assume a fixed stare, while the muscles of the face twitch, and the thumbs are drawn inwards upon the palms of the hands. These nervous symptoms—well known to nurses by the name of inward fits—are of importance, as being sometimes the forerunners of convulsions. The tongue is at first covered with white fur, through which red papillæ project; afterwards it is apt to become pale and clean, or with little patches of fur scattered here and there over the dorsum. In bad cases the whole body has an offensively sour smell. This smell proceeds not only from the breath, but from acidity of all the secretions; the saliva, the perspiration, and the urine being all intensely acid. The cutaneous secretion is, however, seldom in excess; more usually the skin is dry, and is in consequence harsh and rough to the feel, especially at the backs of the arms and the belly. The feet are generally cold, and the child lies with the knees drawn up to the abdomen. The coldness of the feet is no doubt one cause of the griping pains which are so constant in this derangement, for even in healthy infants abdominal pains are frequently excited by coldness of the feet, and cease when these are warmed. During the earlier periods of this disorder the complexion turns slightly yellow from time to time, the yellow tint remaining for some hours or days. Occasionally the skin becomes completely jaundiced. After the complaint has existed for some time a peculiar earthy tint is noticed of the face and whole body, which is very characteristic of chronic abdominal derangement.

If the disorder is primary, and is not soon arrested, a chronic catarrh of the stomach is often set up, the bowels becoming obstinately confined, and the vomiting continuing as a persistent condition. In other cases, again, the derangement may settle principally upon the bowels, leading to a chronic diarrhœa. The most extreme emaciation is often reached through these means, and it may be only after weeks, or even months, of illness that a termination, by recovery or by death, is arrived at.

When the disorder is secondary to some acute disease, or follows a serious operation, the strength is usually so much reduced by the original illness that the child, weakened more and more by the vomiting and diarrhœa, and by his inability to digest any nourishment whatever, soon becomes exhausted. Thrush appears upon the inside of the mouth, and the child sinks and dies. Pneumonia is a not uncommon complication in the later stages of the disease, and, if the strength be much reduced, may exist without manifesting its presence by any of the usual symptoms. There is no cough, and the heat of the body is not appreciably heightened, or if heightened at first, the elevation of temperature soon passes off. This pneumonia usually attacks the bases of both lungs.

The earlier treatment is commenced in this derangement the more readily will the complaint be arrested, for as the strength becomes more and more reduced, and the stomach and bowels become more and more disordered, treatment which in an early stage would be at once attended by improvement loses much of its efficacy, and great difficulty is experienced in making any impression upon the disease.

When the case is seen early, and the symptoms complained of are merely griping and flatulence, with ravenous appetite, unaccompanied by sickness or diarrhœa, careful inquiry should at once be made into the diet and general management of the infant. It should be explained to the parents that the appetite will best be satisfied, not by increasing the quantity of farinaceous matter and the frequency of the meals, but by carefully adapting the food supplied, both in quality and quantity, to the digestive power of the child, so that the nourishment given may be only such as the stomach is able to digest. This may seem a simple and self-evident proposition, but it is one which is constantly forgotten. That a child will be nourished in exact proportion to the amount of food he swallows, and that the more solid the food the greater its nutritive power, are two articles of faith so firmly settled in the minds of many mothers that it is very difficult indeed to persuade them to the contrary. To them wasting in an infant merely suggests a larger supply of more solid food — every cry means hunger, and must be quieted by an additional meal. It is difficult to lay down precise rules for diet in every case of this derangement. This is a matter which can be properly learned only by experience. There are, however, certain plain rules which should always be observed. Of these one of the most important is, that farinaceous food is unsuitable to an infant under the age of three months. Before that age he should be restricted entirely to the breast, supposing that the secretion of milk be of proper quality and be supplied in sufficient quantity. In cases, however, where additional food has to be given on account of the insufficient supply of breast milk, recourse must be had to cow's milk, or to the milk of the ass. If cow's milk be used, it should be diluted with a third part of lime-water, in order to prevent the too firm coagulation of its casein. Even, however, when thus diluted and alkalinized, the cow's milk is sometimes undigested by young infants, who seem to thrive better upon the milk prepared with a very small quantity of arrowroot or baked flour. This scarcely accords with the statement made above, of the unsuitability of such foods to young infants; but an explanation of the seeming contradiction is found in considering the action of the farinaceous food under such conditions. The arrowroot itself probably contributes little, if anything, to the nutrition of the body, but when thus intimately mixed with the cow's milk it has a mechanical action in separating the casein into minute portions. The curd, therefore, coagulates, not in one large clot, but in a multitude of small clots, which are more readily attacked by the digestive juices. It is, however, as has been said, always a risk to give farinaceous food to young infants, and the same object may be as readily effected, and without any danger to the child, by adding a small quantity of isinglass or common gelatine to the diluted milk, in the proportion of one teaspoonful to four ounces.

In older children, brought up upon artificial food, the above symptoms are often complained of, even although the quality of the food with which they are supplied leaves nothing to be desired. In these cases it is the quantity which is in fault; the child is supplied with food largely in excess of his wants or his powers of digestion, and the stomach and bowels revolt against the burden imposed upon them. For an infant of six months old, one, or for a very robust child, two, teaspoonsful of farinaceous food, carefully prepared with milk, and given twice in the day, are as much starchy matter as he is able readily to digest. His other meals should be composed of milk and lime-water, or the milk and water with isinglass, as directed above.

The kind of farinaceous food is of some importance. Different foods vary very much in the proportion of their several constituents, and the albumen, gluten, salts, etc., they contain, are to be considered quite as much as the starchy matter. The very best food is, perhaps, pure wheaten flour slowly baked in an oven till it crumbles into a light grayish powder. This, prepared with milk, and sweetened with milk sugar, forms an admirable morning and evening meal. It may be varied occasionally with other farinaceous articles, but whatever be the food selected, the quantity mentioned must not be exceeded. On alteration in the diet, in accordance with the above rules, a small dose of castor-oil, or rhubarb and soda, to clear out undigested matter from the bowels, and the administration of a little bi-carbonate of soda or potash, with an aromatic to neutralize any remaining acidity and promote digestion, are all the measures that are required at this stage.

If the derangement have gone on to vomiting and purging, with an intensely sour smell from the breath and from the ejected matters, other means must be resorted to. In this case the stomach and bowels are filled with the acid products of fermentation, and the vomiting and diarrhœa are merely the forcible efforts of the alimentary canal to expel its irritating contents. Sedatives to the stomach and astringents to the bowels are here out of place; we shall best cure the derangement by assisting the expulsion, and not by obstructing the exit of the fermenting food. In determining, however, the exact measures to be adopted, the state of the child's strength is an important consideration, and this is best estimated, not by the condition of the pulse, but by the degree of depression of the fontanelle. If the fontanelle is not much hollowed, a teaspoonful of ipecacuanha wine should be at once administered, and should be repeated every ten minutes until vomiting be produced. The acrid matters in the stomach having been thus evacuated, half a teaspoonful of castor-oil should be given after a short interval, to act gently on the bowels, and the child should be allowed nothing but a little cold, thin barley-water, given occasionally, with a teaspoon. At the same time the belly should be kept covered with a hot linseed-meal or bran poultice, and the child warmly wrapped up should be kept perfectly quiet in his little cot.

If the derangement have only existed a short time, the above measures will be usually successful in checking the symptoms, and the child will be found to retain the breast milk, or the milk and water with which he is supplied in small quantities. Any tendency to acid fermentation that may remain should be neutralized by five-grain doses of bi-carbonate of soda, given three or four times a day, and the patient may be allowed to return very gradually to his ordinary diet.

When, however, the derangement is of long duration, or is secondary to a severe operation or to some acute disease, the symptoms are not so easily overcome. Here the weakness, as shown by the depressed fontanelle, will not allow very active measures to be employed, and therefore the accomplishment of our twofold object, viz., of removing already formed acid from the system, and of preventing further fermentation, requires the most careful management. Emetics are here out of the question, for the strength will not bear further reduction, and the administration of such a remedy would be attended by the greatest danger. Our first care should be to endeavor to restore the circulation to the extremities, by placing the feet as high as the knees in hot mustard and water. If the weakness be very great, the whole body may be immersed in a mustard bath as high as the neck. It is of extreme importance in such cases to restore the proper action of the skin, for it is by this means, chiefly, that we hope to effect the escape of acid from the system. On being removed from the bath the infant should be carefully dried; a hot linseed-meal poultice is then to be applied to the belly, and the child, well wrapped in flannel, must be returned to his cot. The warmth of the surface must be kept up by hot bottles placed by his sides, and the feet and legs should be well rubbed at intervals with the hand alone, or with a liniment composed of equal parts of compound soap liniment and the compound liniment of camphor. If the child can bear the motion, frictions with the same embrocation may be used to the whole body; but in cases where the weakness is extreme and the vomiting obstinate, violent retching may be excited by the slightest movement, so that the frictions would have to be discontinued. In such cases the feet and legs should be wrapped in hot flannels on which some flour or mustard has been sprinkled, and the most perfect quiet should be enforced. A napkin must be placed under the chin, to receive all matters ejected from the stomach, and when moistened the cloth must be immediately removed and a clean one applied in its place.

If diarrhœa exist, astringents are not to be employed so long as a sour smell from the breath and evacuations indicates the continuance of fermentation in the stomach and bowels. For a child of a year old, twenty drops of castor-oil can be administered, and will be usually kept down. After its action a simple chalk mixture may be given, or a draught containing five grains of bi-carbonate of soda, with three grains of nitrate of potash, in some aromatic water, three or four times in the day. Half a drop of tincture of capsicum is a valuable addition to each dose of this mixture.

If there be constipation, the bowels must be opened by an enema containing castor-oil, and be kept in regular action by the occasional administration, as required, of one or two drops of a solution of podophyllin in alcohol (a grain to the drachm), or by suppositories of castile-soap placed in the rectum.

The form of nourishment to be given in these cases is of the utmost importance. All matters capable of undergoing fermentation must of course be excluded. Even milk itself, however diluted and alkalinized, can seldom be borne, as it is usually vomited sour and curdled immediately after being taken. Woman's milk is usually well digested, but not always. In some cases it seems to agree as little as the milk of the cow; in others, where the irritability of the stomach is very great, the mere movement of the mouth in the act of sucking may be sufficient to excite a return of the vomiting. If this be found to occur, the breast milk should be given with a teaspoon. In cases where a return to the breast is impracticable, or is not followed by the expected improvement, a good food is whey, made fresh as required, by adding prepared rennet to cow's milk in the proportion of a teaspoonful to the pint. To two tablespoonsful of the whey add one tablespoonful of fresh cream, and dilute with two tablespoonsful of hot water. Of this food small quantities can be given at regular intervals, and care must be taken that it be either hot or cold, but not tepid, as liquid food given in a lukewarm state would be apt to favor a return of the vomiting. Liebig's food for infants, carefully prepared with freely diluted cow's milk, will often be borne; but in very bad cases it is inferior to the diet just described. In addition, the waning powers of life must be supported by five-drop doses of pale brandy, given in a teaspoonful of the food every hour, or even oftener, according to the condition of the fontanelle.

By such measures success is often attained even in the very worst cases of this derangement. The obstinate vomiting is best arrested not by sedatives, but by giving the stomach as much rest as is consistent with supporting nutrition. Of all special drugs, calomel in doses of one-eighth or one-sixth grain, laid dry on the infant's tongue, is, perhaps, the one which is the most generally successful; but our chief reliance should be placed on a careful diet, and on stimulating and hot applications, so as to promote the circulation and encourage the free action of the skin. The existence of cold feet alone would be a sufficient obstacle to the success of any treatment whatever.—*American Journal of Obstetrics, Etc.*

### *Note on the Value of the Wheat Phosphates in Therapeusis.*

By J. S. Hawley, M. D.

The researches of modern physiologists have fully demonstrated the importance of inorganic principles in nutrition. For example, however rich in fibrin and albumen the blood may be, it is not properly constituted, nor can it carry on its life-giving functions, without the presence of the salts of iron, soda, and lime. Among these salts stand pre-eminently the phosphates.

Among the early and successful investigators in this field was M. Mouries, who arrived at the following conclusions:

1st. That phosphate of lime plays a more important part in nutrition than has heretofore been believed. Independently of its necessity as a constituent of bone, this salt maintains that irritability without which there is no assimilation, and consequently no nutrition.

Its insufficiency therefore produces death, with all the symptoms of inanition, while its insufficiency in a less degree produces a series of lymphatic diseases.

2nd. The food consumed in cities is deficient in this respect, and nurses' milk has consequently the same defect. The infant suffers from the deprivation of this element so indispensable to its development and life. Hence one of the causes of the increase of the mortality of infants.

3d. *The addition of this salt, in combination with animal matter, to alimentary substances, obviates one cause of disease and death.*

These conclusions were arrived at as the result of a large number of well directed experiments and careful analyses of alimentary substances. He further demonstrated the existence of a constant relation between the animal heat and the amount of phosphates in the blood, from which he deduces the principle that these salts keep up animal irritability, without which nutrition is impossible.

The following is the testimony of Dr. Tilbury Fox, not merely to the value of the phosphates in nutrition, but of the great superiority of the natural over the artificial phosphates.

"There is something *essentially special in the Organized phosphates, those, in fact, which have been formed by passing through a living organism, as compared with those artificially prepared.*

It is not the *amount* but the *kind* exhibited which produces the good result. In infants' food, and in our bread and flour, the organized phosphates and cerealin (which has a somewhat similar action to pepsin) have been deliberately rejected. *These may be administered medicinally to children and infants when the assimilative function is at fault.* In eruptive diseases of the scalp (which are generally associated with faulty assimilation), in rickets, marasmus, chronic diarrhœa, and impaired nutrition of all kinds, the *Wheat Phosphates* act marvellously. Pallid children pick up tone, color, and flesh; worms disappear, the secretions become healthy, and disease goes."

But it is not altogether necessary to look abroad for written testimony to the value of the organized phosphates in alimentation.

The following is an extract from remarks before the New York County Medical Society, by Prof. A. Jacobi:

"The evils of inanition receive marked illustration in practice among children. As an instance of the chronic starvation of special tissues, might be mentioned rachitis, a disease exhibiting defective nutrition of the osseous and muscular systems. The proportion of phosphate and carbonate of lime (chiefly phosphates) in the bones of infants is 60 to 63 per cent.; while in rachitic children, and particularly in rachitic softening of the cranial bones, it falls as low as 50 and even 20 per cent. *The lack of these elements* is most probably due to excessive elimination; we cannot stop this, and must meet it by an *increased supply.* Experience has shown that *a diet rich in phosphates* will often, without medicine, effect a marked improvement.

The following illustrates, in an important particular, the superiority of the natural over the artificial phosphates:

M. Andrè Lauson, in a paper read at the Academy of Medicine, (*Gazette Médicale*, August 14th, 1869), calls the attention of French surgeons to the fact that to promote osseous growth, the administration of phosphate of lime, either in the shape of hypophosphites, or in that of powdered bone, is unavailing. Several attempts made with these substances have never been successful. It is because their form does not allow of their digestion and assimilation. On the contrary, *earthy phosphates, such as are elaborated by vegetables, are real aliments.* Now, wheaten flour contains but ·4 per cent. of phosphoric acid, and ·02 per cent. of carbonate of lime; in wheat bran, on the contrary, we find 2·5 per cent. of phosphoric acid, and ·11 per cent. of carbonate of lime.

*Pure flour is, then, deprived of its phosphates* to a great extent, and the bread made with it is not the proper aliment for the patients referred to in this article."

The above quotations abundantly exhibit the value of wheat phosphates in nutrition. These phosphates are abundant in the bran of wheat, may be easily extracted and reduced to a flour, and thus become a *medicinal food* which will supplement in an important particular the ordinary food upon which infants and children are fed.

It would be useful, as appears from the above extracts, in all cases of inanition, to the offspring of scrofulous patients, in whatever condition of life, and especially to that large class of illy nourished children of poverty found in asylums and hospitals.

The intelligent practitioner would frequently be relieved from embarrassment in his attempts to nourish his feeble and emaciating patients, by having within his reach an available and convenient form of wheat phosphates.

It may be said that a much simpler method of arriving at this point would be to use unbolted flour for the food of such patients as require a diet rich in phosphates. Doubtless such a course would be judicious and useful to the patient. But most of the foods furnished for children are deprived of phosphates, and the article in question would afford an easy and ready means of restoring them and of regulating the quantity. Besides, it is no easy task to overcome a fixed and universal custom, while it is both easy and convenient to prescribe a remedy in those cases where it is indicated.

Greenpoint, L. I.

*** It may not be without interest to note in this connection and in answer to some of the opponents to the use of the wheaten phosphates in alimentation, that Mr. F. Crace Calvert, in a paper read before the British Association, (*Chemical News*, Am. Rep., Nov., 1869,) has stated that "most of the phosphates contained in wheat are not combined with the organic matter, but are in a free condition." (Ed. Gazette.)

—*Medical Gazette.*

### *Local Treatment of Croup by Lactic Acid.*

A knowledge of the power possessed by lactic acid to dissolve fibrinous exudations induced Dr. Adolph Weber to try it in cases of croup. At first he used it only after the operation of tracheotomy, partly with a view to keep the tracheotomy tubes clean, and partly hoping that the lactic acid might affect the membranes which extended downwards into the bronchi. The results were so favorable in both respects that he proceeded to try it in severe cases of croup before having recourse to tracheotomy. Since then he has not once had occasion to operate, and has not lost a single case of croup. In some very severe cases in which inspiration and expiration were equally obstructed, and the condition of the faucus indicated an abundant fibrinous exudation in the trachea, the difficulty of breathing was completely relieved within seven to ten hours of using this remedy, and two or three days after, no trace of the local affection remained.

During the treatment there was not, as is generally the case, an expectoration of tough membranous sputa, but gradually the whistling, barking inspiration and expiration were replaced by distinct rattling noises; the voice, before quite suppressed, began to assume a hoarse timbre, and considerable quantities of loose, white, frothy phlegm were expectorated during the fits of coughing, until at last the struggle for breath quite ceased, and the disease assumed more the character of a catarrhal affection of the throat.

The treatment consists in the local application of the remedy to the windpipe by means of inhalation. The patient is made to inhale a solution of lactic acid (15 to 20 drops in half an ounce of water) at first every half hour, and afterwards, when the respiration improves, every hour or every two hours a solution of 10 to 15 drops in half an ounce of water.

The inhalation is discontinued as soon as the dyspnœa has subsided, and to promote expectoration chamomile tea is exhibited.

In using the inhalation, care must be taken that the vapor does not affect the eyes or face.

With this treatment was conjoined the internal exhibition of carbonate of soda every half hour or every hour, which was thought to exert a beneficial effect upon the exudation.— Med. Times & Gaz., Jan. 22, 1870.—*Med. News.*

### *Diet in Certain Surgical Cases.*

It is now many years since the extirpation of bone with a view to its reproduction by means of the periosteum was first practiced, and it is now a tolerably frequent operation. In the *Abeille Médicale*, M. André Sanson gives his brother surgeons a hint as to the diet most suitable to patients under such circumstances, so as to hasten their complete recovery. In zootechny, it is by particular kinds of food that the maturity of cattle is hastened. The fodder which is richest in calcareous phosphate is the best suited for the purpose, because that is the chief constituent of bone. The seeds of graminacous, leguminous, and oleaginous plants are given with this view to the young animals, which thus, as it were, become older than their real age. To apply this principle to man is now the question. The food of cattle being, of course, unsuited for the human race, we must examine what alimentary substances fit for it will best answer the purpose of favoring the reproduction of bone. Now beans, peas, lentils, and such like leguminous seeds, contain upwards of 0·85 per cent. of phosphoric acid, and are therefore to be recommended as the best diet in cases of the above description. Bread, which is such an important element of food in France, requires to be specially mentioned. We have often stated in these columns that the whitest flour, although so pleasing to the eye, is far from being the most nutritive. Wheat flour does not contain more than 0·40 per cent. of phosphoric acid, and

0·02 of lime; that of rye, on the contrary, possesses 0·70 of the former, and 0·05 of the latter; but the bran of wheat contains as much as 2·50 of the one and 0·11 of the other; so that flour of the first quality is deprived of the greater part of its phosphate. Hence, patients in whom the regeneration is to be promoted, ought to eat brown bread in preference to white. This is a far better plan than endeavoring to introduce phosphates into the economy in their mineral state.—Galignani.—*Med. Gazette.*

### *The Sulphited Phosphate of Lime.*

Phosphate of lime, in whatever state it may be, dissolves readily in an aqueous solution of sulphurous acid. The solution has the taste and smell of the sulphurous acid, but greatly diminished in intensity. A boiling heat slowly decomposes the solution, sulphurous acid escapes, and a heavy white crystalline precipitate is formed, which, when washed and dried over sulphuric acid, is found to have the formula, $3\ CaO, P^2O^5, SO^2, 2H^2O$. This sulphited phosphate of lime has no smell or taste, and is distinguished from all other sulphites by its stability. Even when heated for several hours up to 266 degrees Fahrenheit, it is not decomposed. But though it withstands the action of the atmosphere indefinitely, it is rapidly oxidized when mixed with the soil. It acts, in that case, as a soluble phosphate of lime, and a trial of several seasons has proved it to be an excellent manure. It has also remarkable antiseptic and disinfecting powers, and its physical properties recommend it strongly for these purposes. It is a clear white powder, which stains and soils nothing, and can be brushed off from carpets and garments, leaving no mark; it is free from smell and taste, and harmless to animal life. Its harmlessness and freedom from taste and smell suggest its being tried in therapeutics, where it may prove a valuable agent.

The solution of phosphate of lime in sulphurous acid also acts as a disinfectant, and in some cases with even more energy than the powder. It may be used to advantage in places where a liquid can be more conveniently applied than a solid.—*Boston Journal of Chemistry.*

### *Nephrotomy.*

On Thursday, Feb. 3, the operation of cutting into the kidney for removal of renal calculus was performed by Mr. Durham, at Guy's Hospital. The theatre was crowded with students and others anxious to see so rare an operation. We believe, indeed, that there is only one instance of its performance recorded, and that was by a surgeon of Venice, upon an Englishman, some hundred and fifty years since. Two or three stones were then extracted, to the great relief of the patient, a urinary fistula remaining in the loin. Owing, doubtless, as much to the uncertainty of the diagnostic signs of renal calculus as to the supposed risks of the procedure, the operation fell into oblivion, until a paper read before the Medico-Chirurgical Society last year, by Mr. Thomas Smith, of St. Bartholomew's Hospital, again brought the subject before the notice of the profession. Although Mr. Durham's bold attempt ended in disappointment, we saw enough to convince us that as far as cutting down upon the kidney is concerned, there is neither great difficulty nor any apparent grave risk in the proceeding. Mr. Durham made his incision along the edge of the erector spinæ, from the pelvis to the eleventh rib, and quickly reached the hilus of the kidney without difficulty, and with little or no loss of blood, but no stone was found, although the symptoms previously manifested had been such as are considered characteristic of stone in the kidney. The hilus of the kidney and the ureter, for the space of an inch and a half, were thoroughly examined, but not opened, no stone being felt, and their general appearance, as well as that of the kidney, being perfectly healthy. So far from the operation having been injurious, five days later the woman expressed herself as being more free from pain than she had been for a long time! Full details of the case will be published whenever it may be considered to have terminated, whatever the termination may be.—Med. Times & Gaz., Feb. 12, 1870.—*Med. News.*

### *Iodine in Intermittent Fever.*

PROFESSOR WILLEBRAND, of Helsingfors, has, in certain forms of the disease, successfully used the following: ℞. Iodine, gramme j; pot. iod., gramms ij; aq. distil, grammes x; M. S. Five drops given in water every two hours. Relapses seldom occur, as in the use of quinine. Iodide of iron, in doses of ten centigrammes, four times a day, for cachexia and anæmia.—*Archives Generales.*

### *Surgery of the Hip-Joint.*

MR. WILLIAM ADAMS has divided subcutaneously the neck of the thigh bone within the capsular ligament, by means of a fine saw. This is a great triumph of subcutaneous surgery, as the case is doing well.—*Med. Press and Circular.*

### *Sir Duncan Gibb*

ATTRIBUTES the diminished longevity of the Jews to the persistent use of olive oil for culinary purposes, resulting in what he terms a "*Sanguineo oleaginous expression,*" pendency of the epiglottis, premature decrepitude, and a tendency to death from congestive disease.—*Med. Bulletin.*

